# Enhanced flame retardancy of polyethylene/magnesium hydroxide with polycarbosilane

**DOI:** 10.1038/s41598-018-32812-5

**Published:** 2018-09-27

**Authors:** Chunfeng Wang, Yongliang Wang, Zhidong Han

**Affiliations:** 1 School of Materials Science and Engineering, Harbin University of Science and Technology, 150040 Harbin, China; 2 Key Laboratory of Engineering Dielectrics and Its Application, Ministry of Education, Harbin University of Science and Technology, 150080 Harbin, China

## Abstract

Polycarbosilane (PCS) was used for surface modification of magnesium hydroxide (MNH) to enhance the flame retardant effectiveness by forming cohesive binding between MgO particles with ceramic adhesive. Chemical interaction and ceramic reaction were revealed between PCS and MNH, which made for a compact, thermal stable and ceramic-like barrier during the combustion of polyethylene (PE). The flame retardancy of PE/MNH/PCS composites was greatly enhanced and a limiting oxygen index (LOI) of 35.0 was achieved at the PCS/MNH ratio of 4/26 in the composite with 30 *wt.*% PCS modified MNH. Such results were superior in terms of high LOI value at low global content of MNH. Thanks to the better shielding effect of the integrated and self-supporting ceramic char, the peak heat release rate (p-HRR) and the total heat release (THR) of PE/MNH/PCS composites with 50 *wt.*% PCS modified MNH were remarkably decreased by 36% and 25% in comparison with PE/MNH with 50 wt.% MNH, respectively. The ceramic reaction between PCS and MNH, the superior thermal stability due to crosslinked PCS and the good barrier function of cohesive ceramic layer play important roles in the effective flame retardant mechanism.

## Introduction

Magnesium hydroxide (MNH) is one of the most universal flame retardant additives for polyethylene (PE), polypropylene (PP), ethylene-vinyl acetate copolymer (EVA) and other polymers^1,2^. MNH acts as an inorganic flame retardant and is considered as an environmental-friendly additive^3,4^. High loading levels of MNH (>50 *wt.*%) are usually required to meet the flame retardant demands due to its low flame retardant efficiency^5^, which leads to poor mechanical properties and processing properties of the resulting materials^6^. A lot of effort has thus been devoted to enhance the flame retardant efficiency of MNH by incorporation of synergistic additives^7–10^ and modification^11,12^. Some good results were delivered in the last decade^13–16^. However, the high global content of flame retardants is essential to meet the strict standard requirements. It is still a big challenge to obtain MNH based inorganic flame retardants to meet the requirements for high flame retardant efficiency even though MNH is one of the most widely used flame retardant additives both in amounts and in application fields.

MNH plays its flame retardant role through the endothermic dehydration between 300 °C and 340 °C^17^. MgO as the decomposition product has few effect in promoting polymer charring and low ability to form effective barrier^18^, both of which are considered key factors in flame retardancy of polymers^19–21^. Taking these factors into account, cohesive structure between MgO particles to form an effective barrier would be helpful in improving the flame retardancy of MNH. To the knowledge of the authors, reports on such methods are limited and their flame retardant mechanism is a meaningful issue.

In the recent years, polymeric ceramic precursors were widely investigated to prepare ceramics, such as polysiloxane^22^, polysilazane^23^ and polycarbosilane (PCS)^24^. Inspired by this, PCS, a ceramic precursor, is thus used to modify the surface of MNH and expected to be a good adhesive between MgO particles. In this way, a ceramic barrier would be built by binding MgO particles during ceramic process. Attempts were made to find a competitive synergistic method to improve the flame retardant efficiency of MNH and reduce its global content.

PE is used as flame retardant target here because of two reasons. One reason is that PE leaves no residue after degradation and the charring effect of PCS can be clearly investigated^25,26^. The other reason is that PE shows high heat release during combustion and the barrier effect of the char can be clearly revealed^27^. For more, PE is one of the most widely used thermoplastics and commonly used in wire and cable industries that require high flame retardant properties. According to the results reported, MNH content was as high as 58.3 *wt.*% to obtain a LOI of 30.0 for LLDPE/EAA^13^, while a LOI of 26.0 was achieved for LDPE with 48 *wt.*% MNH and 4 *wt.*% MMT^14^. In our laboratory work, a LOI of 27.8 was achieved when 27 *wt.*% MNH and 3 *wt.*% PCS were directly applied into LDPE. The LOI was further increased to 32.0 when MNH was surface modified with PCS at the same content. Accordingly, we proposed a practical method to obtain MNH based flame retardant of high effectiveness by combining the ceramic precursor with the surface modification technique. It would be an effective way for PCS modified MNH to form a compact, cohesive and thermally stable ceramic layer, which can provide a better shielding effect to enhance the flame retardancy of PE and decrease the heat release during combustion. The mechanistic investigation would provide reference results in designing inorganic flame retardants of high flame retardant effectiveness.

## Results and Discussion

### Flame retardant properties of the composites

**Figure 1:**
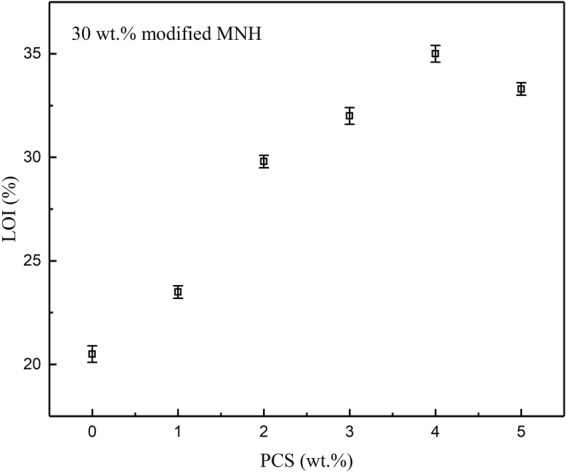
LOI values of PE/MNH/PCS composites with different contents of PCS at the global MNH/PCS content of 30 *wt.*%.

**Figure 2:**
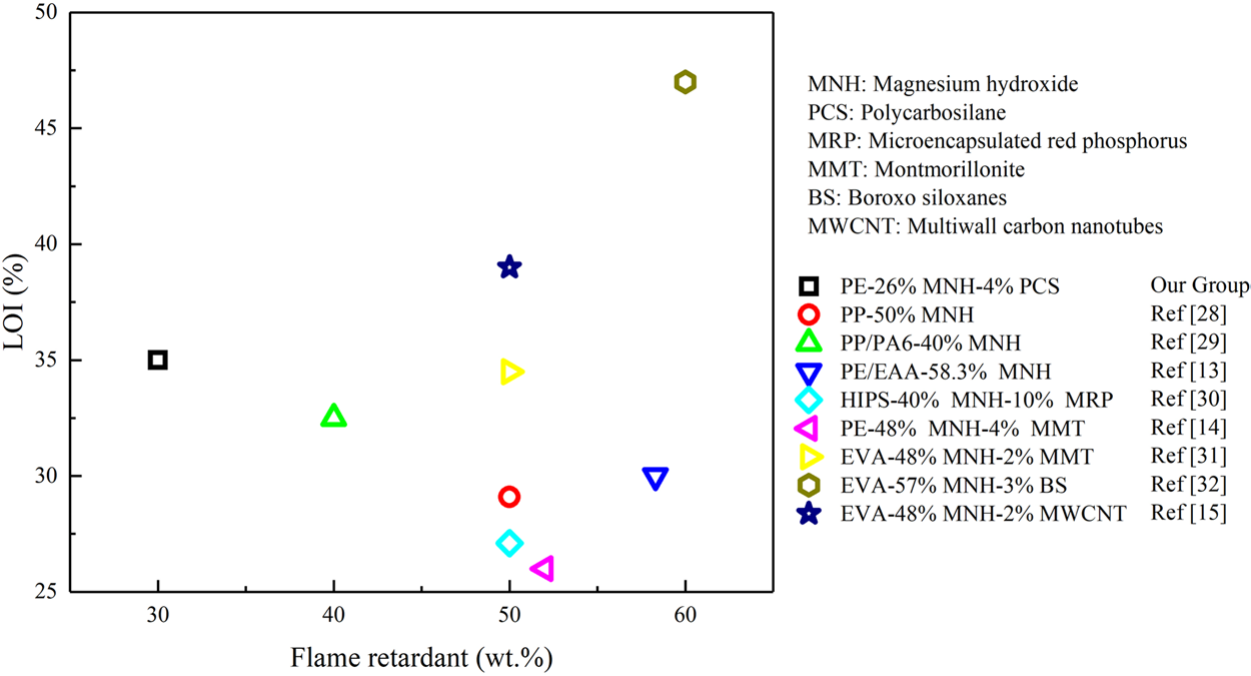
LOI results of the composites with MNH flame retardants reported in the last decade.

Figure 1 shows the LOI values of PE/MNH/PCS composites at the global MNH/PCS content of 30 *wt.*%. As the content of PCS increases from 0 to 4 *wt.*%, the LOI value of the composites increases almost linearly. The composite with 4 *wt.*% PCS shows the LOI value of 35.0, which is 14.5 higher than PE/MNH (20.5). However, the LOI value decreases from 35.0 to 33.5 when the PCS content increases from 4 to 5 *wt.*%. The reasonable explanation is that the addition of PCS induced higher heat release from the matrices. As shown in Fig. S1, the total heat release (THR) of PE/PCS increases by 37% in comparison with PE. Higher PCS content would contribute to more heat release, which impairs the advantages of the ceramic effect of PCS and deteriorates the flame retardant properties of the composites. A synergistic effect between MNH and PCS is hence revealed according to the greatly enhanced flame retardancy of the composites. Such synergistic effect is very remarkable in comparison with the results reported in the literatures^13–15,28–32^ as shown in Fig. 2. The better effectiveness of MNH/PCS is evidenced by the LOI value of 35.0 at the global content as low as 30 *wt.*%, which exceeds most of the reported composites at high content of 50 *wt.*%.

### Thermal degradation of the composites

**Figure 3:**
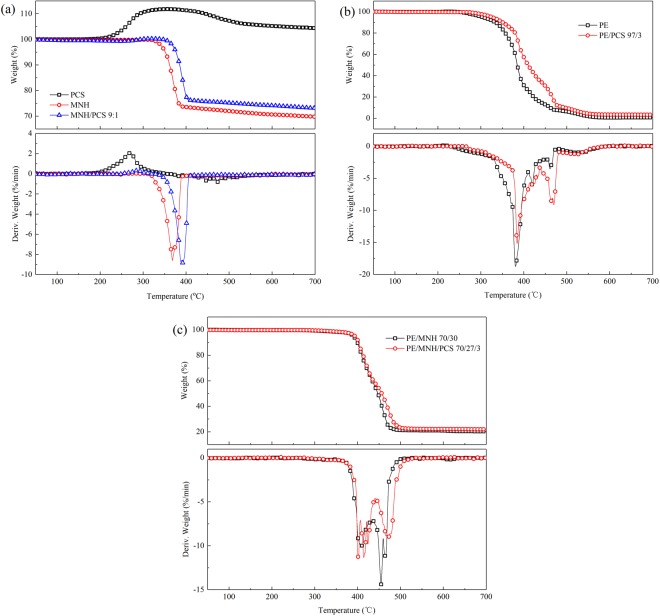
TG and DTG curves of (**a**) PCS, MNH and MNH/PCS; (**b**) PE and PE/PCS; (**c**) PE/MNH and PE/MNH/PCS in air.

**Figure 4:**
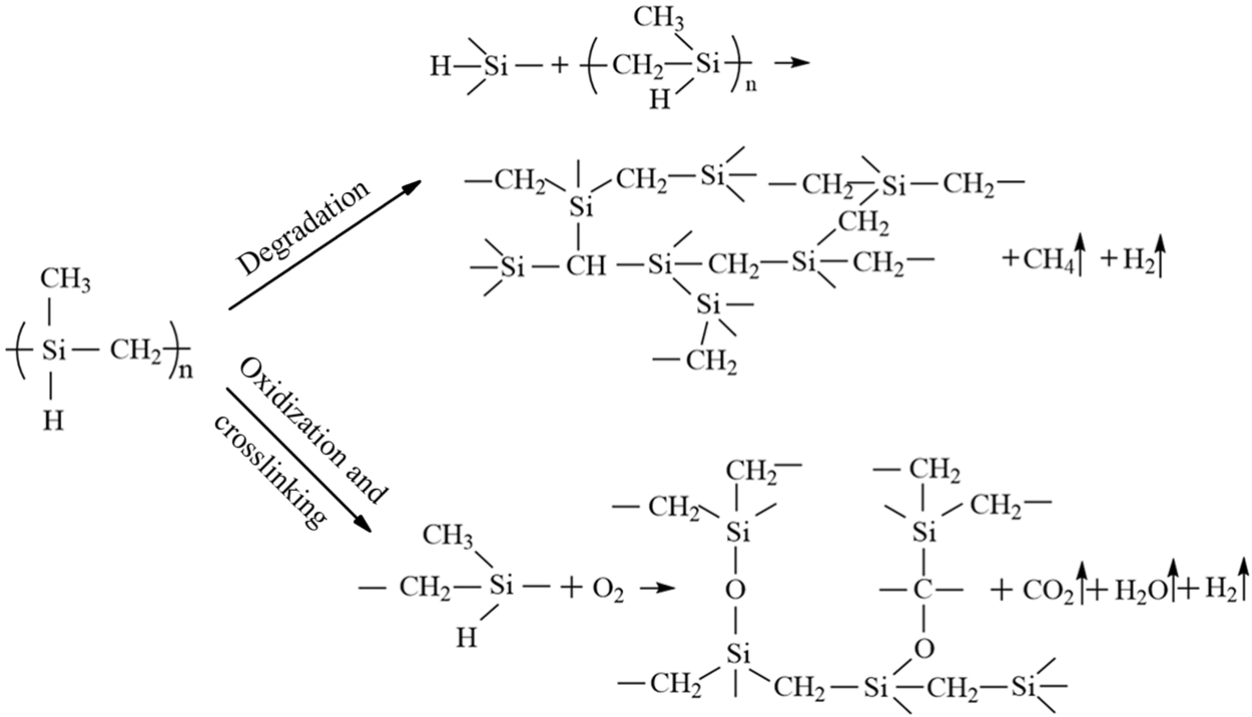
The degradation and oxidation reaction of PCS.

As shown in Fig. 3(a), PCS shows a weight increase of 12% from 200 to 350 °C and a weight loss of 7% from 350 to 600 °C during thermal degradation in air. While thermally heated in air atmosphere, PCS undergoes oxidation crosslinking and thermal degradation as depicted in Fig. 4. PCS chains are connected to form 3D network by oxidation and reaction with Si-H groups. Oxidation crosslinking of PCS happens around 200 °C and gives rise to a remarkable weight gain and improved thermal stability^33^. The non-crosslinked PCS chains undergo thermal degradation and contribute mainly to the weight loss before 700 °C due to the high thermal stability of crosslinked PCS^34^. The residue of PCS at 600 °C is about 105%, indicating the strong oxidation crosslinking of PCS. The combustible gas products, such as CH_4_ and H_2_, give rise to the high heat release during the combustion of PCS. Ceramization process of PCS as precursor of silicon carbide occurs at higher temperature than 700 °C^35^.

A comparison is made between MNH and MNH/PCS at the ratio of 9:1 in Fig. 3(a). MNH/PCS shows about 24 °C higher temperature than MNH both at 5% weight loss (T_5_) and 10% weight loss (T_10_). The maximal weight loss rate (Rmax) of MNH/PCS is similar to MNH, and the corresponding temperature (Tmax) is higher than MNH. The residue of MNH/PCS at 600 °C is 3.4% higher than MNH. Table S1 in supporting information gives the TG data. As a result, the thermal stability of MNH is improved by the modification with PCS, which also increases the residue of MNH/PCS and slows down the decomposition rate. The weight of MNH/PCS increases slightly during the temperature range between 280 and 320 °C due to the oxidation crosslinking of PCS and thus improves the thermal stability of MNH/PCS^36^.

The TG curves of PE/PCS shift to higher temperature in comparison with PE, as shown in Fig. 3(b). The thermal stability of PE/PCS is apparently improved by showing higher T_5_ and T_10_ by 23 and 17 °C, respectively. The degradation of PE chains is depressed and occurs at higher temperature, which can be proved by the increased Tmax by 5 °C, the decreased Rmax of PE/PCS by 16% and the increased weight loss rate at higher temperature (470 °C). The residue of PE/PCS at 600 °C is 2.9% higher than PE.

MNH/PCS exerts strong influences on the degradation behaviors of flame retardant composites. As shown in Fig. 3(c), PE/MNH/PCS shows 4 °C higher T_5_ and 1.3% more residue at 600 °C than PE/MNH. As for PE/MNH/PCS, three peaks are observed in the DTG curves around 400 °C while only one wide peak for PE/MNH, which can be ascribed to char forming and rebuilding at high temperature. As formed char is proved to be effective in protecting the matrix and suppressing the degradation of macromolecular chains by showing the lower weight loss rate and the higher peak temperature between 450 °C and 500 °C. Furthermore, only one peak is observed for PE/MNH/PCS while two peaks for PE/MNH, which indicates the better barrier effects of the char of PE/MNH/PCS. These phenomena can be interpreted by the formation of an effective char layer, which contributes greatly to stabilize the PE chains and suppress thermal degradation.

### Ceramization of the flame retardant

**Figure 5:**
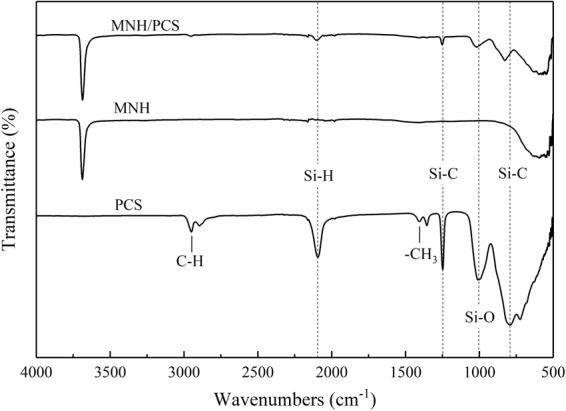
FT-IR spectra of PCS, MNH and MNH/PCS at room temperature.

Figure 5 illustrates the FT-IR spectrum of MNH/PCS(9:1), which shows the typical absorption bands of PCS and MNH^37,38^. The bands at 2950 and 2895 cm^−1^ are ascribed to the stretching vibrations of C-H. The band at 2100 cm^−1^ is ascribed to the stretching vibration of Si-H. The bands at 1355 cm^−1^ and 1250 cm^−1^ are ascribed to the deformation vibrations of Si-C from Si-CH_2_-Si and Si-CH_3_, respectively. The band at 1020 cm^−1^ is ascribed to the stretching vibration of Si-O which originates from Si-O-Si due to the oxidation of PCS at 1005 cm^−1^ and moves to higher wavenumbers in MNH/PCS. According to the band shift of Si-O to higher wavenumbers, the hydrogen bond interaction was formed between MNH and PCS. The band at 830 cm^−1^ is ascribed to the stretching vibration of Si-C which moves from 790 cm^−1^ due to the formation of Si-O-C-Si during the oxidation of PCS. The bands in the spectra of PCS, MNH and MNH/PCS were collected in Table S2. After treated at 500 °C, the spectrum of MNH/PCS illustrates the absorption bands from Si-C, Si-O and Mg-O in Fig. S2. Accordingly, the interaction between MNH and PCS can be interpreted in the hydrogen bond, which makes it possible for the ceramization process at high temperature.

**Figure 6:**
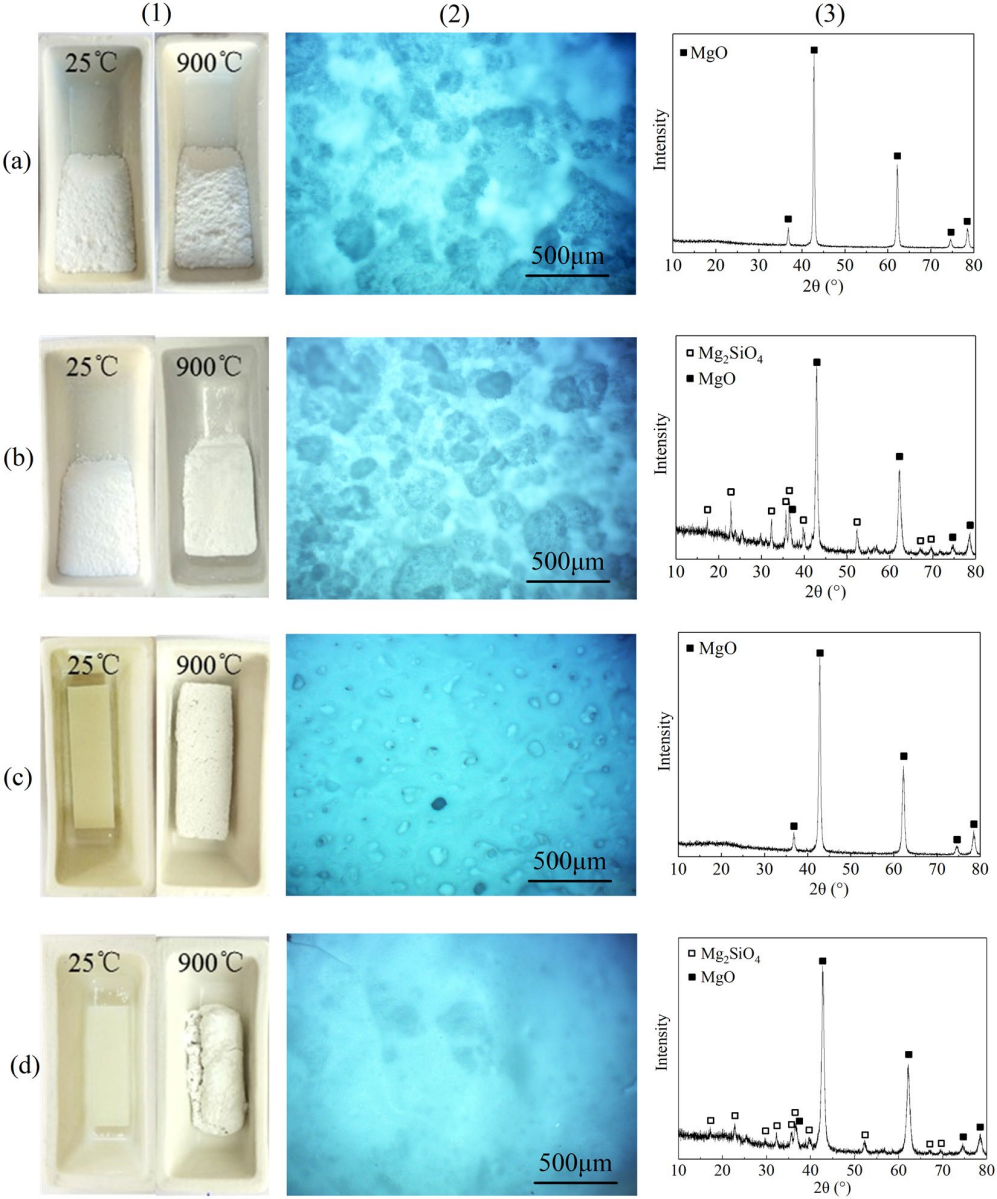
Digital photos (1), optical micrographs (2) and XRD patterns (3) of MNH (**a**), MNH/PCS (**b**), PE/MNH (**c**) and PE/MNH/PCS (**d**) after heat treatment at 900 °C for 1 h.

The ceramization process between MNH and PCS is evidenced by XRD pattern and optical microscopy of the sintered product at 900 °C, as shown in Fig. 6. MNH/PCS undergoes volume shrinkage and forms ceramic-like block after sintered at 900 °C for 1 h, which is in sharp contrast with the powder-like product after sintering of MNH. Although optical pictures show no remarkable micro-morphological changes between sintered products of MNH and MNH/PCS, XRD pattern of sintered MNH/PCS reveals the emerging phase of Mg_2_SiO_4_, which strongly demonstrates the ceramic reaction between PCS and MNH. Silicon carbide is hardly observed due to its amorphous structure. Mg_2_SiO_4_ is also revealed in the residue of PE/MNH/PCS. After sintered, PE/MNH keeps the volume shape and forms the powder-like residue while the ceramic-like residue of PE/MNH/PCS shows cracks due to the volume shrinkage. The residue of PE/MNH/PCS shows smooth and compact surface while that of PE/MNH presents ragged and porous surface. Thus, the cracked structure of sintered PE/MNH/PCS can be attributed to volume shrinkage and inner stress due to the ceramic reaction between MNH and PCS and the gas releasing during the degradation of PE and MNH.

Accordingly, the ceramic reaction between MNH and PCS produces Mg_2_SiO_4_. Since PCS was applied on the surface of MNH for modifications, Mg_2_SiO_4_ can act as adhesive between MgO powders and form a self-supporting ceramic block. Such ceramic process also works for PE/MNH/PCS and gives rise to a compact and smooth barrier on the surface of the residue. The barrier would be helpful in improving the flame retardancy of PE/MNH/PCS due to its good thermal stability and effective function in depressing heat and mass transfer between the condensed phase and the gas phase. Instead, the porous structure of PE/MNH residue indicates the poor barrier effect in gas releasing during the degradation of PE/MNH.

### Combustion behaviors of the composites

**Figure 7:**
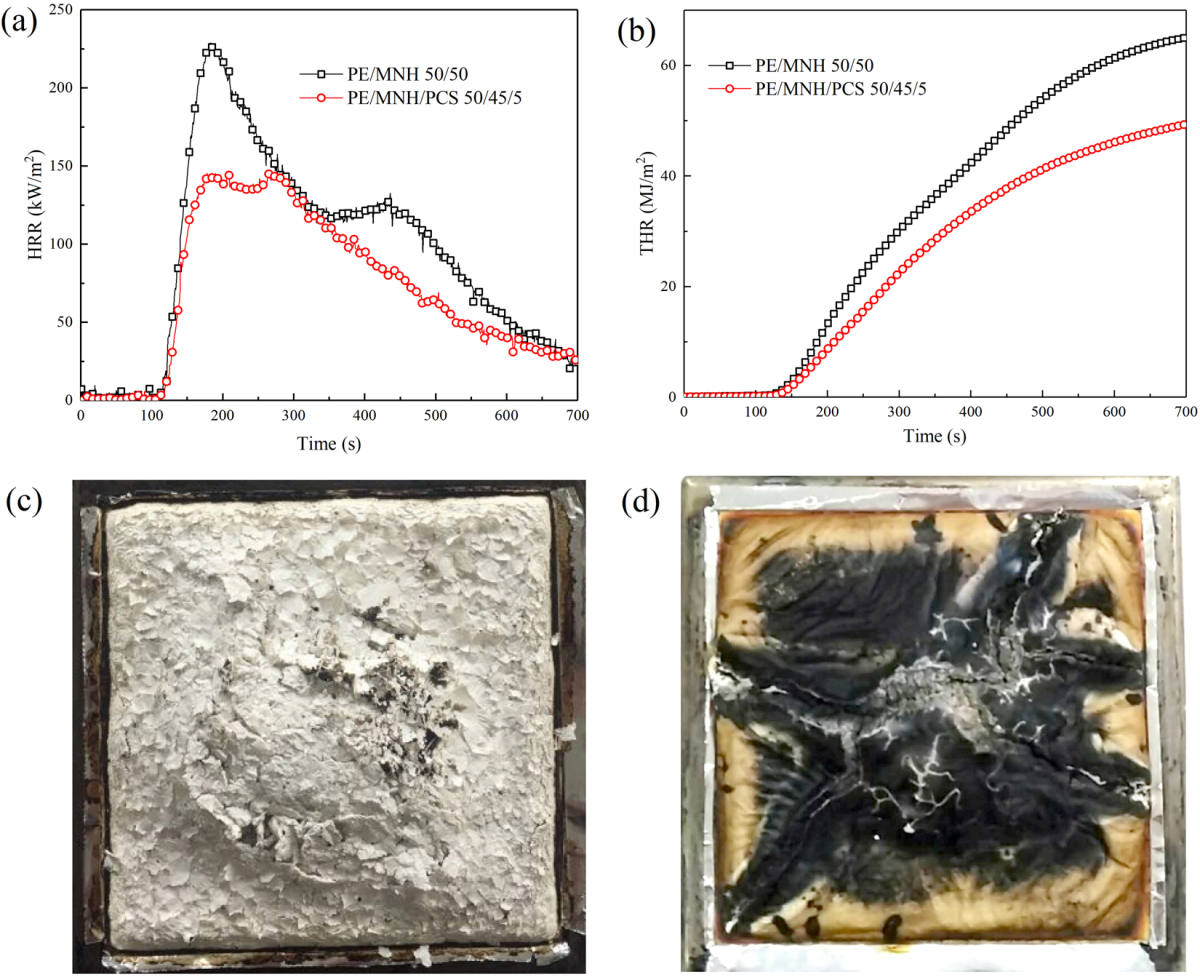
HRR curves (**a**), THR curves (**b**) and the digital photos of the char residue after CONE test of PE/MNH (**c**) and PE/MNH/PCS (**d**).

Cone calorimeter test is considered as one of the best way in assessing combustion behaviors of materials^39^. More information can be collected^40^, among which heat release rate (HRR) and total heat release (THR) are of great importance^41^. When the global content of flame retardant is 50 *wt.*%, the combustion behaviors of PE/MNH and PE/MNH/PCS composites are compared in Fig. 7. The p-HRR of PE/MNH/PCS in Fig. 7(a) is greatly reduced to 143.2 kW/m^2^ in comparison with 228.8 kW/m^2^ of PE/MNH, a decrease by 37.4%. Furthermore, THR of PE/MNH/PCS in Fig. 7(b) is decreased by 25%. The residue of PE/MNH/PCS after combustion in Fig. 7(d) shows very different morphology from that of PE/MNH in Fig. 7(c), which evidences the formation of ceramic char with compact and self-supporting characteristics and contributes to the better shielding effects. Such char barrier is effective in suppressing the mass loss during combustion. As shown in Fig. S3, the mass loss of PE/MNH/PCS is significantly inhibited during combustion in contrast to that of PE/MNH. The mass residue of PE/MNH/PCS is increased by 35% than PE/MNH. The disappearance of the shoulder peak in the HRR curves of PE/MNH/PCS further supports the good thermal stability and barrier function of the ceramic char. Consequently, the heat release and the mass loss are greatly depressed in PE/MNH/PCS.

### Mechanism discussion

**Figure 8:**
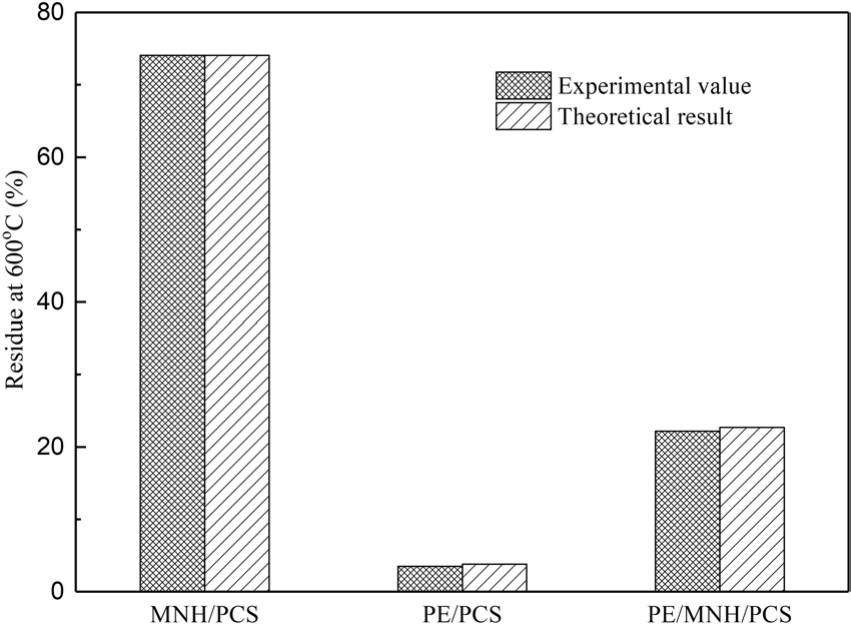
Theoretically calculated residues and experimental results of MNH/PCS, PE/PCS and PE/MNH/PCS.

**Figure 9:**
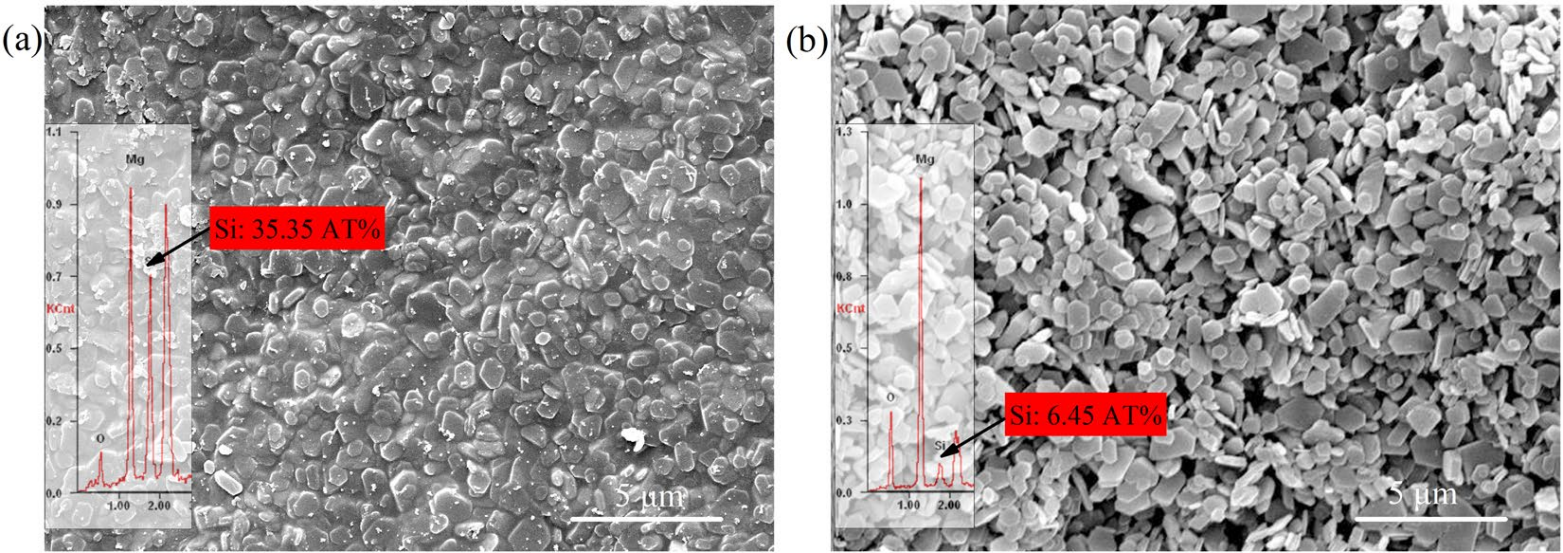
SEM micrographs and EDS spectra of the residue of PE/MNH/PCS composite after Cone test. (**a**) The surface; (**b**) the inner.

The ceramic reaction (in Fig. 6) was revealed and the ceramic product of Mg_2_SiO_4_ was considered as adhesive for MgO particles, which played an important role in forming thermal stable, self-supporting and compact barrier (in Fig. 7). MNH/PCS, PE/PCS and PE/MNH/PCS showed superior thermal stability (in Fig. 3) due to the good thermal stability of crosslinked PCS. However, no charring effect of PCS can be observed while comparing the theoretically calculated residues with the experimental results in Fig. 8. A smooth and compact morphology in Fig. 9(a) was observed at the surface of PE/MNH/PCS residue, which evidences the good adhesive structure of particles. Instead, the inner particles below the surface show poor cohesiveness in Fig. 9(b) and the morphology is similar to that of PE/MNH.

Meanwhile, the surface residue was found to be abundant in Si element in comparison with the inner part, which indicates the surface migration or accumulation of PCS during the combustion (Fig. 9). On the other hand, a three-dimensional structure formed during the degradation and oxidation crosslinking of the molecule chains of PCS (Fig. 4), which resulted in a protective layer on the surface together with MNH. As illustrated in Fig. 7, the protective layer functioned as the good barrier to heat and mass transfer, which kept the heat and oxygen from the inner part. PCS below the protective layers underwent thermal degradation instead of oxidation crosslinking due to the anoxic environment, which would be another reason that the less amount of Si element inside. Such phenomena provide further evidences for the good barrier functions of ceramic layers by the interactions between PCS and MNH.

**Figure 10:**
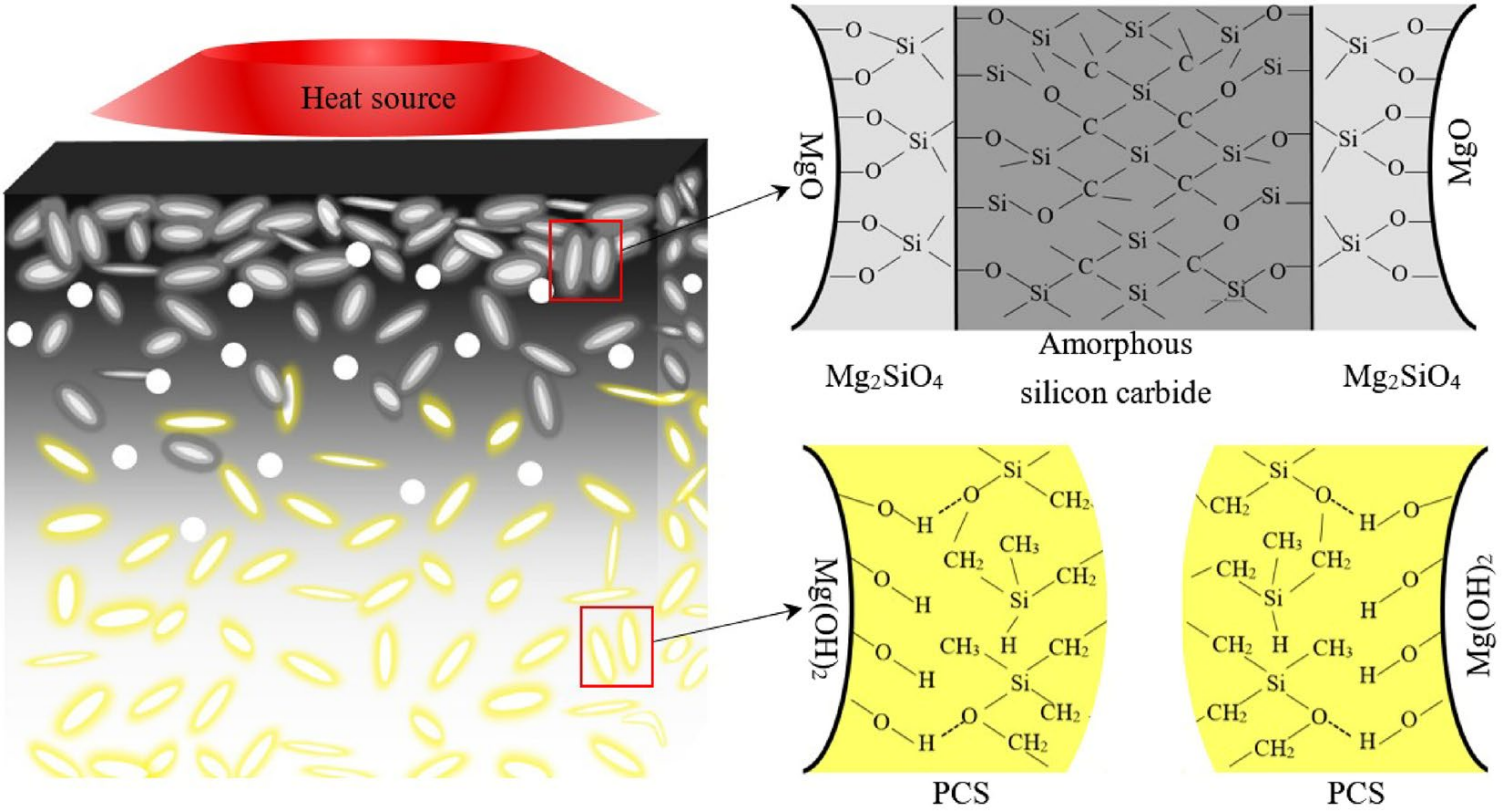
The schematic diagram of char formation.

Consequently, three aspects are proposed in the flame retardant mechanism of PE/MNH/PCS: ceramic reaction between PCS and MNH, superior thermal stability due to crosslinked PCS, effective barrier function of cohesive ceramic layer. The mechanism is schematically illustrated in Fig. 10.

## Conclusions

A practical method was proposed to obtain MNH based flame retardant of high effectiveness by combining the ceramic precursor with the surface modification technique, which overcame the non-cohesive characteristic of MgO and made for a self-supporting, integrated, cohesive ceramic char. The results of FTIR, XRD, SEM and EDS strongly supported the function of PCS as a ceramic precursor in forming ceramic-like barrier. The following conclusions would be helpful in understanding the ceramic process and the flame retardant mechanism.

1. The interaction between PCS and MNH was built through hydrogen bond during surface modification, which made it feasible to achieve an integrated structure by the formation of Mg_2_SiO_4_ interlayer between amorphous silicon carbide and MgO, resulting in a compact, thermal stable and ceramic-like barrier during the combustion.
2. The preferable thermal stability of PE/MNH/PCS was attributed to the formation of a three-dimensional structured ceramic layer due to the crosslinking effect of PCS. Compared with inner part, the abundant of Si element in surface residue indicated the surface migration of PCS and the accumulation of Si during the combustion.
3. No charring effect of PCS was observed. An effective barrier of cohesive MgO particles with ceramic adhesive was the reason for flame retardant enhancement of the composites. The results revealed the good shielding effect in heat and mass transfer.

Consequently, PCS modified MNH was proved to be of high efficiency in flame retardant PE. When the content of MNH/PCS was 30 *wt.*%, the LOI of PE/MNH/PCS composites was as high as 35.0, which was superior to the results reported in the literatures. When the content of MNH/PCS was 50 *wt.*%, the p-HRR and the THR of PE/MNH/PCS in comparison with PE/MNH were decreased by 36% and 25%, respectively.

## Methods

### Materials

Low density polyethylene (LDPE, 2426H, MI = 1.9 g/10 min) was from BASF-YPC Co., Ltd. Magnesium hydroxide (MNH, 5-C) with the particle size of 2-5 μm was from Dandong Songyuan Chemicals Co., Ltd. Polycarbosilane (PCS) was from Suzhou Sailifei Ceramic Fiber Co., Ltd. Xylene was from Tianjin Fuyu Fine Chemical Co., Ltd. All the materials and reagents were used as received.

### Modification of MNH

PCS modified MNH (MNH/PCS) was prepared at different MNH/PCS ratios as shown in Table 1. Typically, 10 g PCS was dissolved in 10 ml Xylene to make the PCS solution. 90 g MNH was mixed with the PCS solution by a high-speed mixer (2000 rpm) for 1 min. MNH/PCS was obtained after drying the mixture in an oven at 80 °C for 12 h. According to the results of particle size distribution (LA-920, HORIBA) and SEM in Fig. S4a and b, no obvious changes were observed in the particle size and the morphology of MNH and PCS modified MNH.

### Preparation of flame retardant composites

PE based flame retardant composites (PE/MNH/PCS) were prepared according to the formulations in Table 1 by using HAPRO rheometer (Harbin Hapro Electric Technology Co., Ltd.). Typically, the rheometer was preheated to 140 °C, and PE pellets were added into the chamber. After melt mixing for 2 min, MNH/PCS was added and mixed for 10 min to obtain PE/MNH/PCS composites. All samples were hot pressed at 150 °C under 10 MPa for 5 min. Good dispersion of MNH and PCS modified MNH were found in Fig. S4c and d.

**Table 1:** The formulations of flame retardant composites.

Sample	PE (*wt.*%)	MNH/PCS	
		MNH (*wt.*%)	PCS (*wt.*%)
PE/PCS 97/3	97	—	3
PE/MNH 70/30	70	30	—
PE/MNH/PCS 70/29/1	70	29	1
PE/MNH/PCS 70/28/2	70	28	2
PE/MNH/PCS 70/27/3	70	27	3
PE/MNH/PCS 70/26/4	70	26	4
PE/MNH/PCS 70/25/5	70	25	5
PE/MNH 50/50	50	50	—
PE/MNH/PCS 50/45/5	50	45	5

### Characterization

Fourier transform infrared (FTIR) spectra were recorded by using infrared spectrophotometer (FTIR iS10, Nicolet) with a resolution of 0.4 cm^−1^. Thermogravimetric analysis (TGA) was performed in air flow at a heating rate of 10 °C/min from 50 to 800 °C by using a TG analyzer (NETZSCH, STA 499 F3). Limiting oxygen index (LOI) was tested using a LOI tester (JF-3, Nanjing Jiangning Analytical Instrument Factory, Inc.) according to ASTMD 2863. Cone calorimeter test was performed by using an FTT cone calorimeter (Fire Testing Technology, East Grinstead) on a specimen (100 × 100 × 3 mm^3^) wrapped with aluminum foil at the external heat flux of 35 kW/m^2^ according to ISO 5660-1. The morphology of char residue after cone test was observed using a field-emission scanning electron microscopy (SEM, FEI200). The structure of sintered products was analyzed by X-ray diffraction (XRD, X′Pert PRO, PANalytical, Netherlands) in reflection mode with Cu Kα radiation at 40 kV with 40 mA current (*λ* = 1.5406 Å). All diffraction patterns were obtained over the 2θ range of 5-80° at a scanning rate of 8°/min. Optical morphology was observed by a polarizing microscope (UPT200i, Chongqing photoelectric instrument Co., LTD). The samples in crucible were observed after sintered.

## Electronic supplementary material

Supporting information

## Data Availability

All data included in this study are available upon request by contact with the corresponding author.
