# Supplementary material for: Enhanced flame retardancy of polyethylene/magnesium hydroxide with polycarbosilane
Source: Sci Rep. 2018 Sep 27;8:14494. doi: 10.1038/s41598-018-32812-5 (PMC6160472; doi:10.1038/s41598-018-32812-5)
Supplement: Supplementary file 1 — Supporting information [file 41598_2018_32812_MOESM1_ESM.pdf]

Supporting Information

Enhanced flame retardancy of polyethylene/magnesium hydroxide with polycarbosilane

Chunfeng Wang [1], Yongliang Wang [1], Zhidong Han [1,2]*

[1] School of Materials Science and Engineering, Harbin University of Science and Technology, 150040, Harbin, China

[2] Key Laboratory of Engineering Dielectrics and Its Application, Ministry of Education ,Harbin University of Science and Technology, 150080 Harbin, China.

* Corresponding author:

Address: School of Materials Science and Engineering, Harbin University of Science and Technology, 150040, Harbin, China

Tel: +86-13936337636

E-mail: zhidong.han@hrbust.edu.cn

Table S1 TG data in air

Sample	T_5 (°C)	T_{10} (°C)	T_{max} (°C)	R_{max} (%/min)	Residue at 600 °C (%)
PCS	260	292	268	+2.1	105.2
MNH	352	362	368	-8.5	70.7
MNH/PCS 9/1	376	385	392	-8.8	74.1
PE	303	334	381	-18.0	0.6
PE/PCS 97/3	326	351	384	-15.1	3.5
PE/MNH 70/30	390	399	455	-14.4	20.9
PE/MNH/PCS 70/27/3	394	402	402	-11.4	22.2

Table S2 Bands in the FT-IR spectra of PCS, MNH and MNH/PCS

Bond	Vibration mode	Band wavenumbers (cm^{-1})		
		MNH	PCS	MNH/PCS
O-H	Stretching	3700		3700
C-H	Stretching	-	2950, 2895	2950, 2895
Si-H	Stretching	-	2100	2100
C=O	Stretching	-	1637	1637
C-H	Deformation in CH_3	-	1400	
Si-C	Deformation in Si-CH_2-Si	-	1355	
Si-C	Deformation in Si-CH_3	-	1250	1250
Si-O	Stretching in Si-O-Si	-	1005	1020
Si-C	Stretching in Si-O-C-Si	-	790	830
Si-C	Stretching in Si-CH_2-Si	-	790	-
Si-C	Stretching in Si-CH_3	-	720	-
Mg-O	Stretching	590	-	590

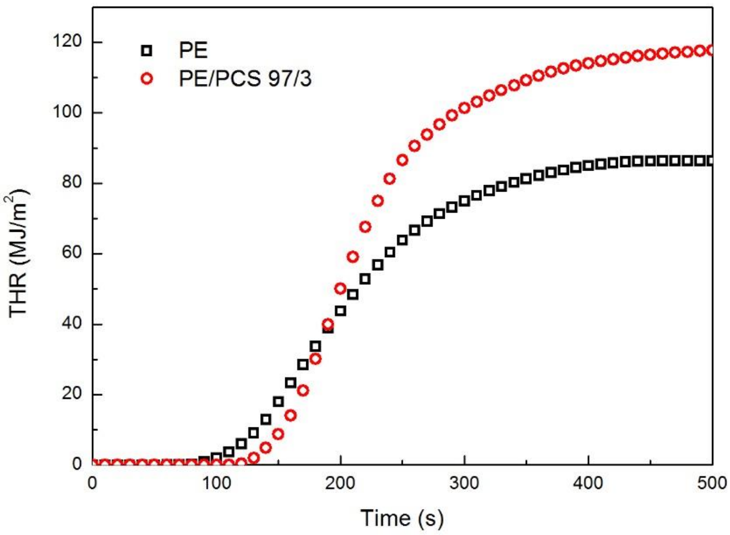

Figure S1 The total heat release (THR) of PE and PE/PCS

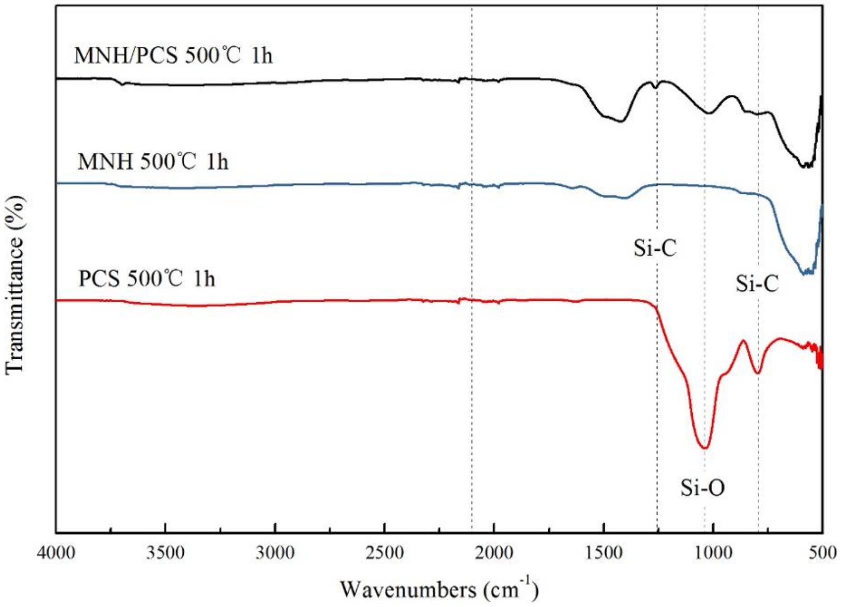

Figure S2 FT-IR spectra of PCS, MNH and MNH/PCS treated at 500 ºC for 1h

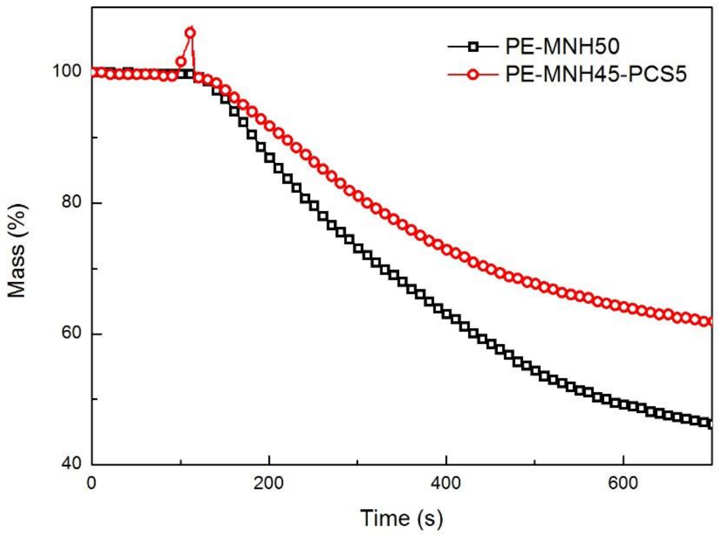

Figure S3 Mass loss curves of PE/MNH and PE/MNH/PCS composites

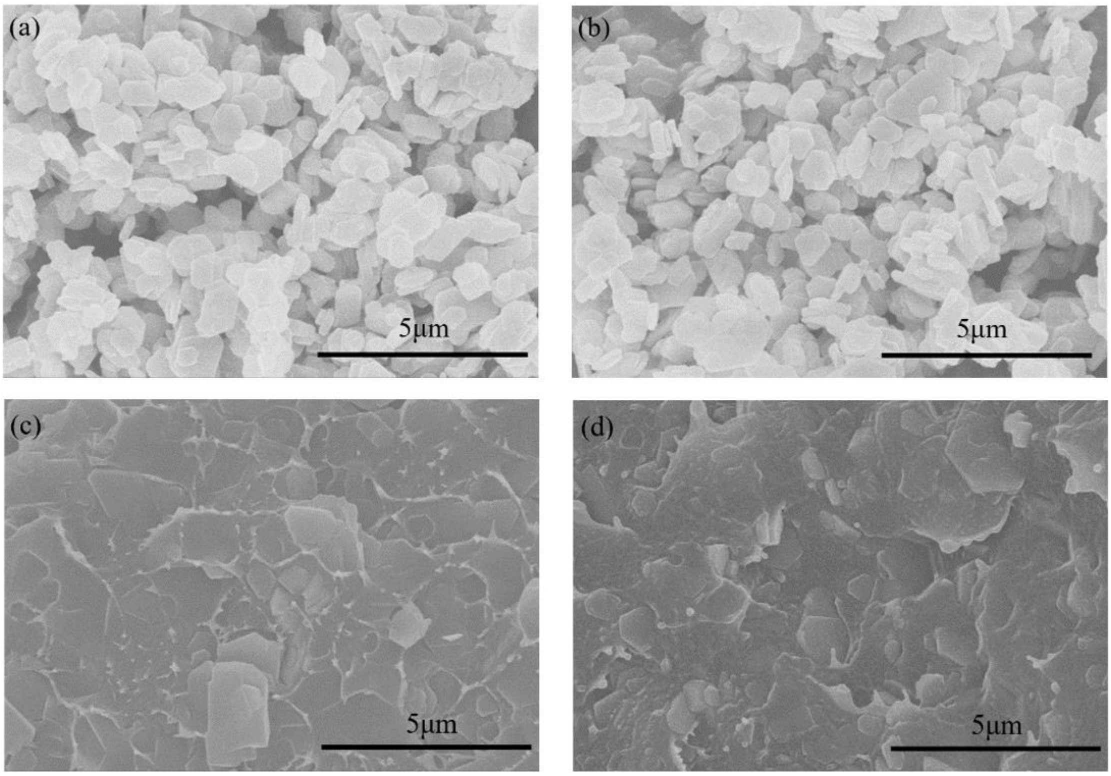

Figure S4 SEM micrographs of MNH (a), MNH/PCS (b), PE/MNH (c) and PE/MNH/PCS (d)
